# SMARCB1-Deficient Skull Base Chondrosarcoma with 12p Duplication Presenting as Somatic-Type Malignancy Arising from Metastatic Seminoma

**DOI:** 10.1007/s12105-023-01610-5

**Published:** 2024-01-18

**Authors:** Prokopios P. Argyris, Bindu Challa, Swati Satturwar, Kyle K. VanKoevering, Paul E. Wakely

**Affiliations:** 1 Division of Oral and Maxillofacial Pathology, The Ohio State University College of Dentistry, Postle Hall, Room 2191 305 W. 12th Ave, Columbus, OH 43210 USA; 2 Department of Pathology, The Ohio State University Wexner Medical Center, James Cancer Hospital and Solove Research Institute, Columbus, OH USA; 3 Department of Otolaryngology-Head and Neck Surgery, The Ohio-State University Wexner Medical Center, Columbus, OH USA

**Keywords:** Chondrosarcoma, Head and neck, Somatic-type malignancy, Testicular germ cell tumor, Seminoma, SMARCB1, IDH1/2, Isochromosome 12p

## Abstract

Somatic-type malignancy (STM) can occur infrequently within a primary or metastatic testicular germ cell tumor (TGCT) and is associated with dismal prognosis and survival. STM with chondrosarcomatous features is exceedingly rare and head and neck involvement has not been previously documented. A 39-year-old white man presented with nasal obstruction and epistaxis. Imaging disclosed a 6.9-cm expansile tumor involving the nasal cavity and skull base with intraorbital and intracranial extension. The histopathologic properties of the tumor were compatible with chondrosarcoma, grade II-III. Immunohistochemically, malignant cells were strongly and diffusely positive for S100 and epithelial markers, and showed loss of SMARCB1 expression. *IDH1/2* mutations were not detected. Following whole-body PET scan, a 7.0-cm left testicular mass was discovered and diagnosed as seminoma with syncytiotrophoblastic cells, stage pT3NXM1b. Extensive retroperitoneal, mediastinal, and supraclavicular lymphadenopathy was also noticed. Histopathologic examination of the left supraclavicular lymph node revealed metastatic seminoma. By FISH, most metastatic nodal seminoma cells harbored 1 to 4 copies of isochromosome 12p, while the chondrosarcoma featured duplication of 12p. Presence of a malignant TGCT with disseminated supradiaphragmatic lymphadenopathy, the unique immunophenotypic properties of the skull-based chondrosarcoma and lack of *IDH1/2* aberrations with gain of 12p strongly support the diagnosis of STM chondrosarcoma arising from metastatic TGCT. The patient did not respond to chemotherapy and succumbed three months after diagnosis. Although exceedingly uncommon, metastasis to the head and neck may occur in patients with TGCT. This case of STM chondrosarcoma demonstrated divergent immunophenotypic and molecular characteristics compared to “typical” examples of head and neck chondrosarcoma. High index of suspicion is advised regarding the diagnosis of lesions that present with otherwise typical histomorphology but unexpected immunohistochemical or molecular features.

## Introduction

The chondrosarcoma family represents a heterogeneous group of malignant osseous neoplasms which, by definition, produce a cartilaginous (chondroid) matrix and most commonly occur in the pelvis, femur, humerus, and ribs. Overall, chondrosarcoma accounts for approximately 4% of all sarcoma cases [1, 2] and 11% of all primary bone malignancies [3]. Chondrosarcoma of the head and neck is rare, comprising only 1–12% of all human chondrosarcoma tumors, and shows a slight predilection for men in their fourth-to-sixth decade [2, 4]. Sites of involvement within the head and neck include the sinonasal tract, gnathic bones, larynx, and base of the skull, while pain, swelling and nasal obstruction represent the most common clinical findings [3, 5]. Several histopathologic subtypes of head and neck chondrosarcoma are recognized including conventional, periosteal, dedifferentiated, and clear cell, with the conventional subtype predominant in the maxillofacial bones [2, 5, 6]. At the molecular level, hot spot mutations of the isocitrate dehydrogenase 1 and 2 genes (*IDH1/2*) generally characterize approximately 65% of head and neck chondrosarcoma tumors [4].

Also, tumors with chondrosarcomatous differentiation have been reported rarely in relationship to somatic malignant transformation developing in testicular germ cell tumors (TGCTs) [7–10]. Somatic-type malignancy (STM) occurs in 2.5 – 8% of TGCTs, exclusively in postpubertal individuals aged 15–68 years (mean age: 33 years) [11, 12]. These most commonly arise in association with a concomitant or prior teratoma, or mixed GCT with a teratomatous component [7, 13, 14]. A smaller subset of STMs appear to derive from non-teratomatous TGCTs including yolk sac tumor [15, 16], and rarely, seminoma [13, 17]. STM can develop within primary testicular malignancies or metastatic tumors to lymph nodes, commonly retroperitoneal and mediastinal, or other organs following cisplatin-based chemotherapy treatment [18, 19]. Development of STM in metastatic GCT severely affects prognosis and patient survival, while the prognostic significance of STM occurring within the primary tumor is less understood. Deciphering whether a metastatic STM originates from TGCT or represents *de novo* malignancy involving other organs or tissues may be challenging, particularly when conventional GCT is not identified within the biopsy specimen [14].

Examples of primary or metastatic TGCT with STM exhibiting overt chondrosarcomatous features are exceedingly rare in the English literature [7–10] and, to the best of our knowledge, involvement of the head and neck region has not been previously documented. Herein, we report the clinicopathologic, immunohistochemical (IHC) and molecular characteristics of a skull base chondrosarcoma which strongly support the diagnosis of STM arising from a metastatic TGCT.

## Case Presentation

### Clinical Findings at Initial Presentation

**Fig. 1:**
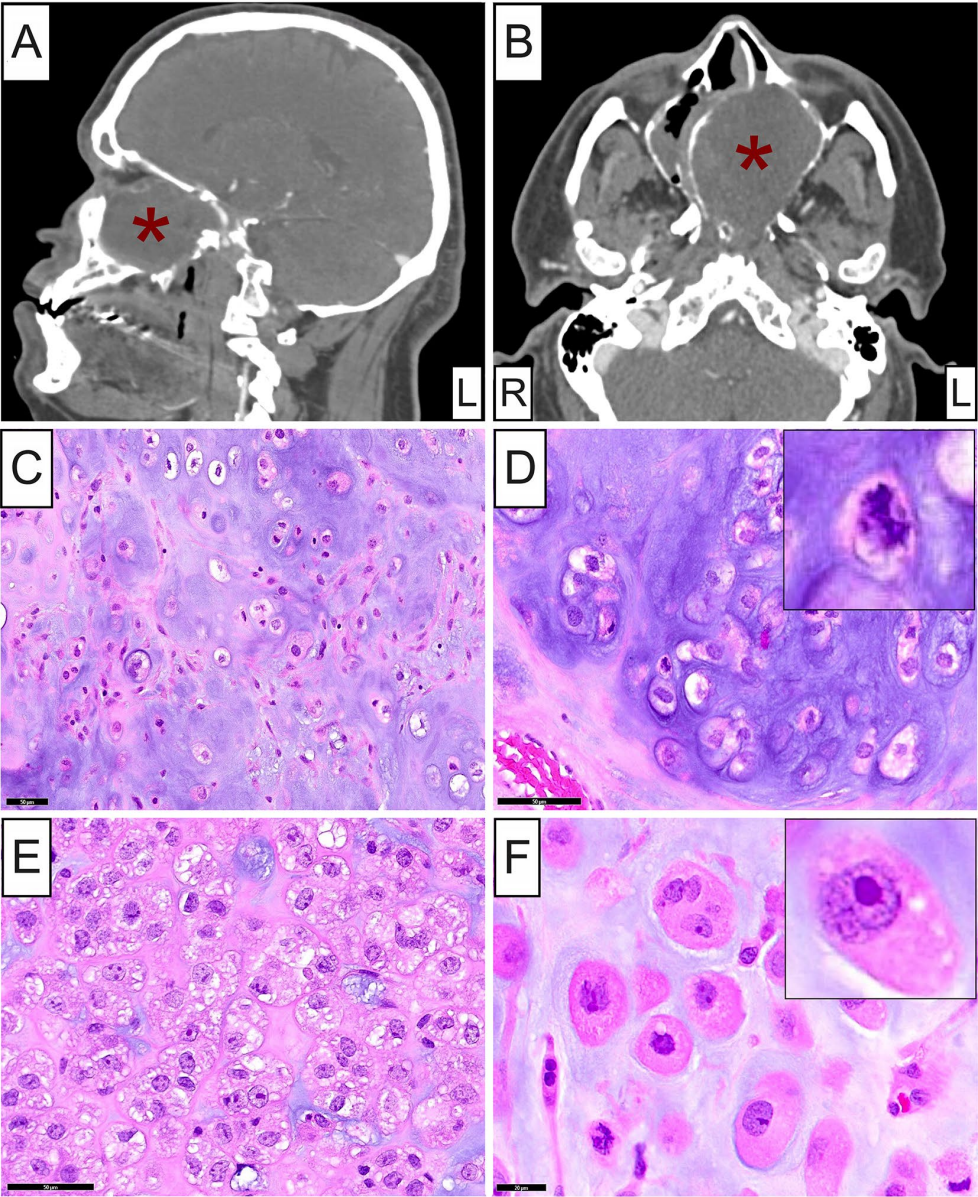
Clinical and histopathologic characteristics of the skull-based chondrosarcoma. Sagittal **A** and axial **B** plane CT scan images showing a 6.9 x 5.3 x 5.0 cm, expansile mass (**red asterisk**) centered in the nasal cavity causing nasal septum erosion, destruction of all nasal turbinates with extension into the maxillary sinuses, as well as intraorbital and intracranial involvement. **C** and **D** Low- and medium-power photomicrographs depicting a modestly cellular cartilaginous neoplasm with multilobular architecture and increased mitotic activity (**inset**). **E** and **F** Medium- and high-power photomicrographs of different areas of the chondrosarcoma tumor showing increased cellularity and clusters of large atypical malignant cells immersed in varying amounts of eosinophilic or amphophilic chondromyxoid stroma. Acidophilic macronucleoli (**inset**), multinucleation, intranuclear cytoplasmic pseudoinclusions and atypical mitoses were present

A 39-year-old white man presented with a 6-month history of gradually worsening nasal obstruction and congestion with occasional episodes of epistaxis. Previous ophthalmologic evaluation due to visual disturbances had confirmed bilateral optic disc edema. MRI and CT scan imaging disclosed a 6.9 × 5.3 × 5.0 cm, expansile, heterogeneous mass centered in the nasal cavity, extending into the anterior ethmoidal cells (Fig. 1A). The mass demonstrated aggressive clinical characteristics including nasal septum erosion, destruction of all nasal turbinates and extension into the maxillary sinuses with adjacent bony remodeling, thinning and dehiscence (Fig. 1B). Intraorbital extension with adjacent remodeling of the lamina papyracea and deformity of the middle rectus muscles was observed, as was intracranial extension with dehiscence of the anterior cranial fossa floor, including the cribriform plate and fovea ethmoidalis (Fig. 1A and B). Severe mass effect on the postchiasmatic optic nerves was appreciated. Destruction of the sphenoid wing and pterygoid processes with involvement of the foramen rotundum, vidian canals and pterygopalatine fossa was also seen along with involvement of the sella and nasopharyngeal extension. Inferiorly, the tumor abutted the hard palate without evidence of erosion. The clinical and radiologic features of the lesion raised concerns for chondrosarcoma or chordoma and an incisional biopsy was performed. The patient, however, developed acute left vision decline which prompted urgent surgery, total resection of the sinonasal mass, and skull base reconstruction with optic nerve decompression. Vision quickly recovered in the postoperative setting.

### Histopathologic and IHC Characteristics, and IDH1/2 Mutation Status of the Skull Base Neoplasm

Microscopic examination revealed a bone-infiltrative cartilaginous neoplasm with a predominantly multilobular architecture and modest cellularity (Fig. 1C and 1D). The lacunar spaces contained round, oval-to-polygonal cells with rich eosinophilic, optically clear or vesicular cytoplasm and distinct cell borders, surrounded by basophilic matrix (Fig. 1C and 1D). Other areas of the tumor featured markedly increased cellularity with clusters of large atypical, round-to-ovoid and, rarely, elongated cells immersed in varying amounts of eosinophilic or amphophilic chondromyxoid stroma (Fig. 1E and 1F). Neoplastic cells showed prominent nuclear pleomorphism with individual nuclei ranging from small, round-to-ovoid, with smooth nuclear contours and evenly distributed dense chromatin (Fig. 1D) to larger, ovoid or irregularly shaped with open chromatin and discernible, one or multiple, acidophilic macronucleoli (Fig. 1E and 1F, **inset**). Intranuclear cytoplasmic pseudoinclusions and multinucleation were frequently present (Fig. 1F), as were mitotic figures, including atypical ones (Fig. 1D, **inset**, and 1F). Focally, in the more cellular areas of the lesion, a few osteoclast-type multinucleated giant cells interspersed with the malignant cells were seen. Evidence of osteoid or immature bone formation was not observed. The above histopathologic findings were compatible with conventional type chondrosarcoma, grade II-III.

**Fig. 2:**
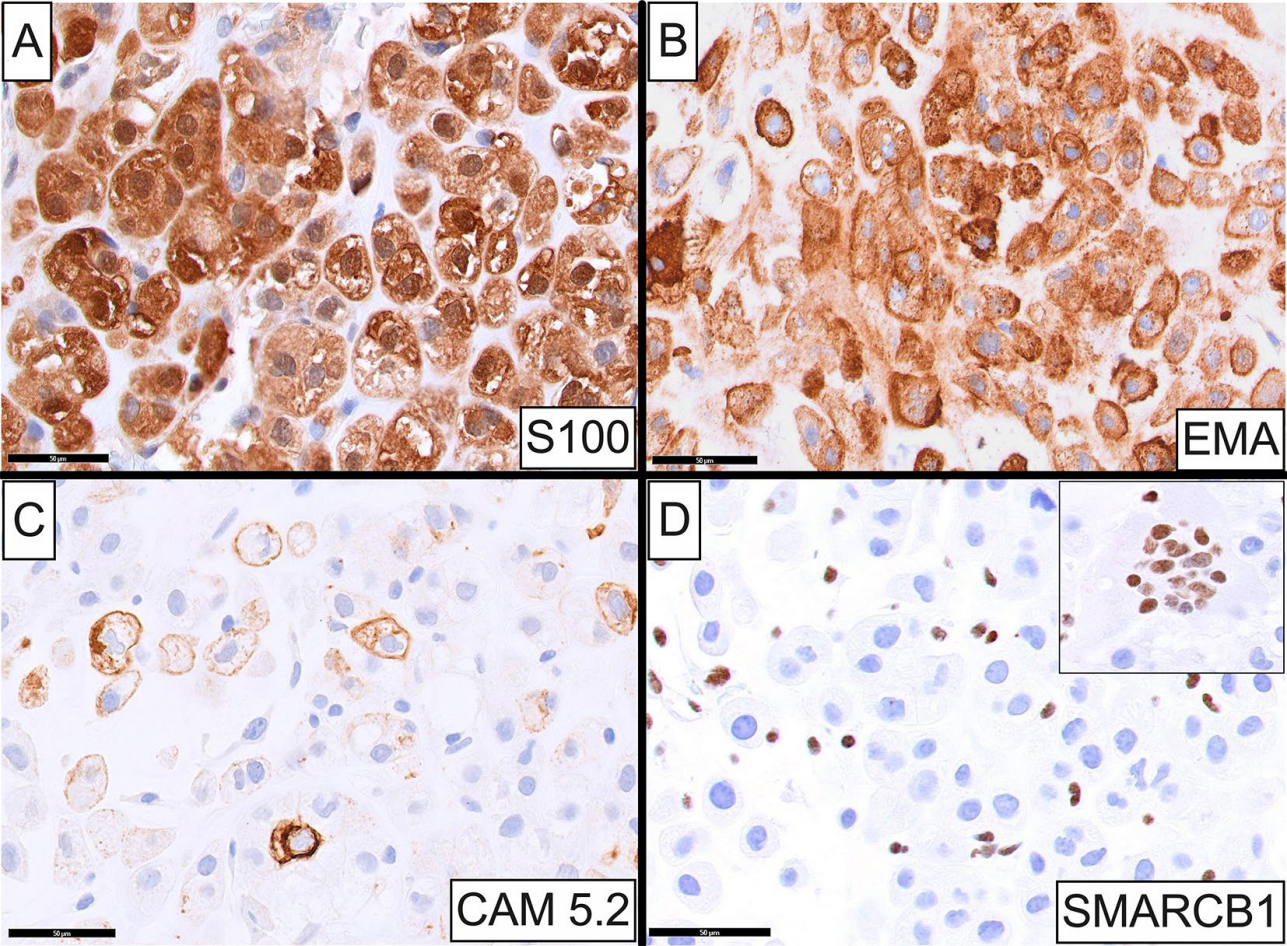
Immunophenotypic profile of the somatic-type malignancy chondrosarcoma. Neoplastic cells were strongly and diffusely positive for S100 **A** and EMA **B**, and focally immunoreactive with CAM 5.2 **C**. SMARCB1 (INI1) expression was lost in malignant cells **D** but retained in background endothelial cells and osteoclasts **(inset)**

Immunohistochemically, chondrosarcoma cells were strongly and diffusely positive for S100 (both nuclear and cytoplasmic staining; Fig. 2A), vimentin, epithelial membrane antigen (EMA; Fig. 2B), and focally immunoreactive with CAM 5.2 (Fig. 2C) and cytokeratin (CK) AE1/AE3. Interestingly, SMARCB1 (INI1) expression was lost in malignant cells (Fig. 2D) but was retained in background endothelial cells and osteoclasts (Fig. 2D, **inset**). Lesional cells were uniformly negative for brachyury, CK MNF116, high molecular weight CK, desmin, SOX10, OCT3/4 and SALL4.

No mutations were detected by pyrosequencing at codon 132 of *IDH1* or codon 172 of *IDH2* on genomic DNA extracted from the chondrosarcoma tumor.

### Additional Clinico-Radiologic Findings and Diagnostic Work-Up

**Fig. 3:**
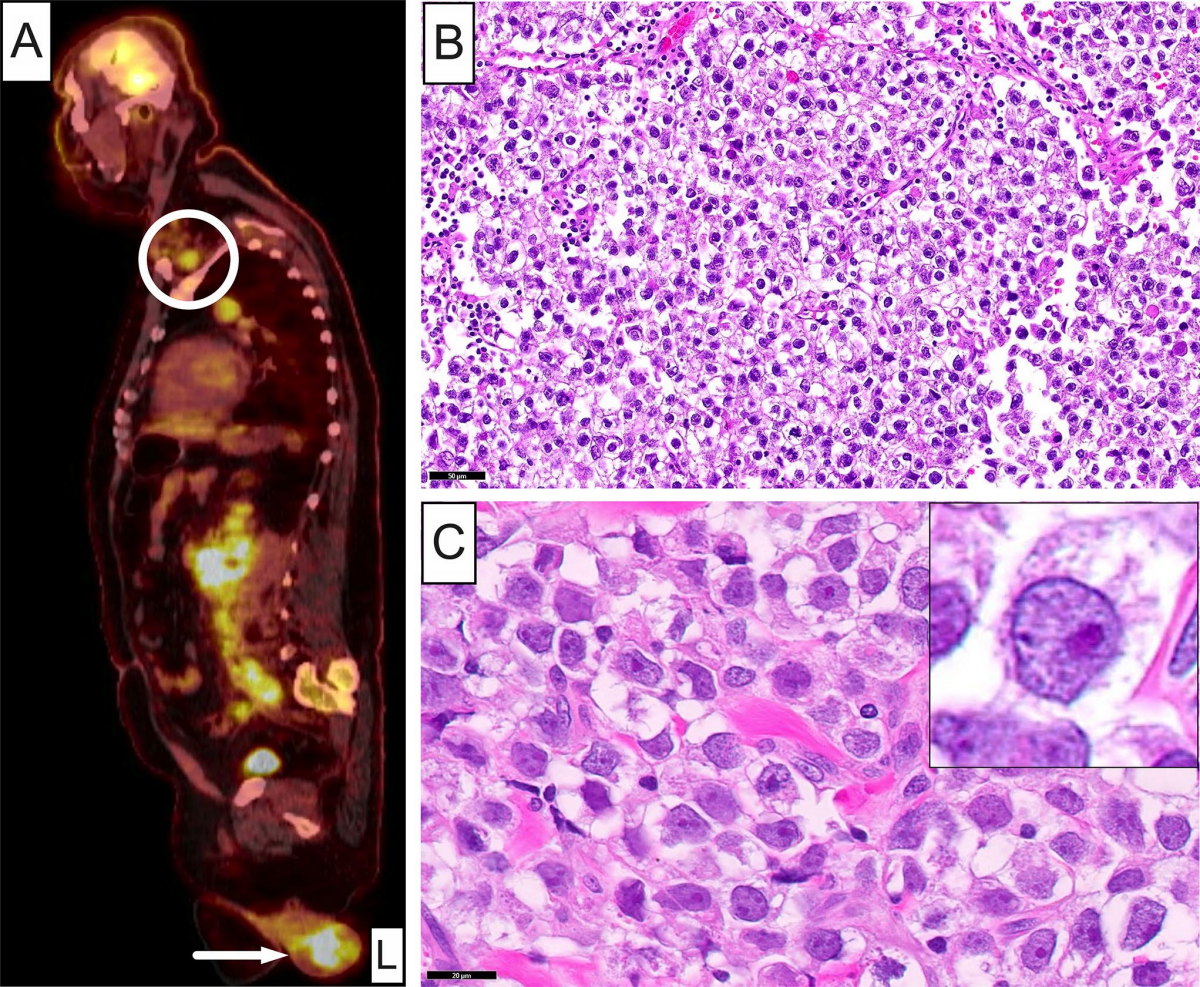
Clinical and histopathologic characteristics of the testicular germ cell tumor. **A** Whole-body PET scan of the patient revealing an intense (max SUV = 12.1), ill-defined, heterogeneous, mass-like activity within the scrotum (**arrow**). The scrotal mass measured 7.0 x 6.4 x 4.7 cm and was associated with the left testicle. Extensive, hypermetabolic, left supradiaphragmatic lymphadenopathy was also noticed, including Virchow’s node (**circle**). **B** and **C** Low- and high-power photomicrographs of the testicular seminoma showing sheets of round to polygonal cells with optically clear, pale or lightly eosinophilic granular cytoplasm and well-delineated cytoplasmic membrane borders. The enlarged nuclei were centrally located or slightly eccentric, round-to-ovoid or angulated, with coarse-to-finely granular chromatin and one or more prominent acidophilic nucleoli (**inset**)

Subsequent whole-body PET scan disclosed mild FDG uptake within the surgical bed with extensive hypermetabolic left supraclavicular lymphadenopathy extending into the subpectoral and anterior mediastinal regions, suspicious for metastatic lymph node involvement (Fig. 3A). Furthermore, marked hypermetabolic retrocrural, retroperitoneal and left periaortic adenopathy, as well as bilateral iliac chain adenopathy were noticed. The pulmonary parenchyma, liver, spleen, and bowel were uninvolved. Notably, the patient also presented with intense (max SUV = 12.1), ill-defined, heterogeneous, mass-like activity within the scrotum (Fig. 3A, **arrow**). The scrotal mass measured 7.0 × 6.4 × 4.7 cm and was associated with the left testicle. Laboratory results showed markedly elevated levels of lactate dehydrogenase and β-human horionic gonadotropin (β-HCG), but normal alpha-fetoprotein. A radical inguinal left orchiectomy was performed along with core needle biopsy of the left supraclavicular (Virchow’s) lymph node.

Histopathologically, the testicular lesion was composed of an infiltrative, uniform, population of malignant cells organized in diffuse sheets with interspersed variably sized fibrous septa (Fig. 3B). In other areas of the tumor, a nodular, nested, or pseudoglandular architecture was seen. Individual cells were round to polygonal with optically clear, pale or lightly eosinophilic granular cytoplasm and well-delineated cytoplasmic membrane borders (Fig. 3B and 3C). Nuclei were enlarged, centrally located or slightly eccentric, round-to-ovoid or angulated, with coarse-to-finely granular chromatin, one or more prominent acidophilic nucleoli, and an irregularly thickened nuclear membrane (Fig. 3C and **inset**). Scattered syncytiotrophoblastic cells were present as was extensive geographic necrosis, patchy lymphocytic infiltration and lymphovascular invasion. A diagnosis of seminoma with syncytiotrophoblastic cells, stage pT3NXM1b, was rendered. No teratomatous component or other histologic types were appreciated in any of the examined histopathologic sections.

**Fig. 4:**
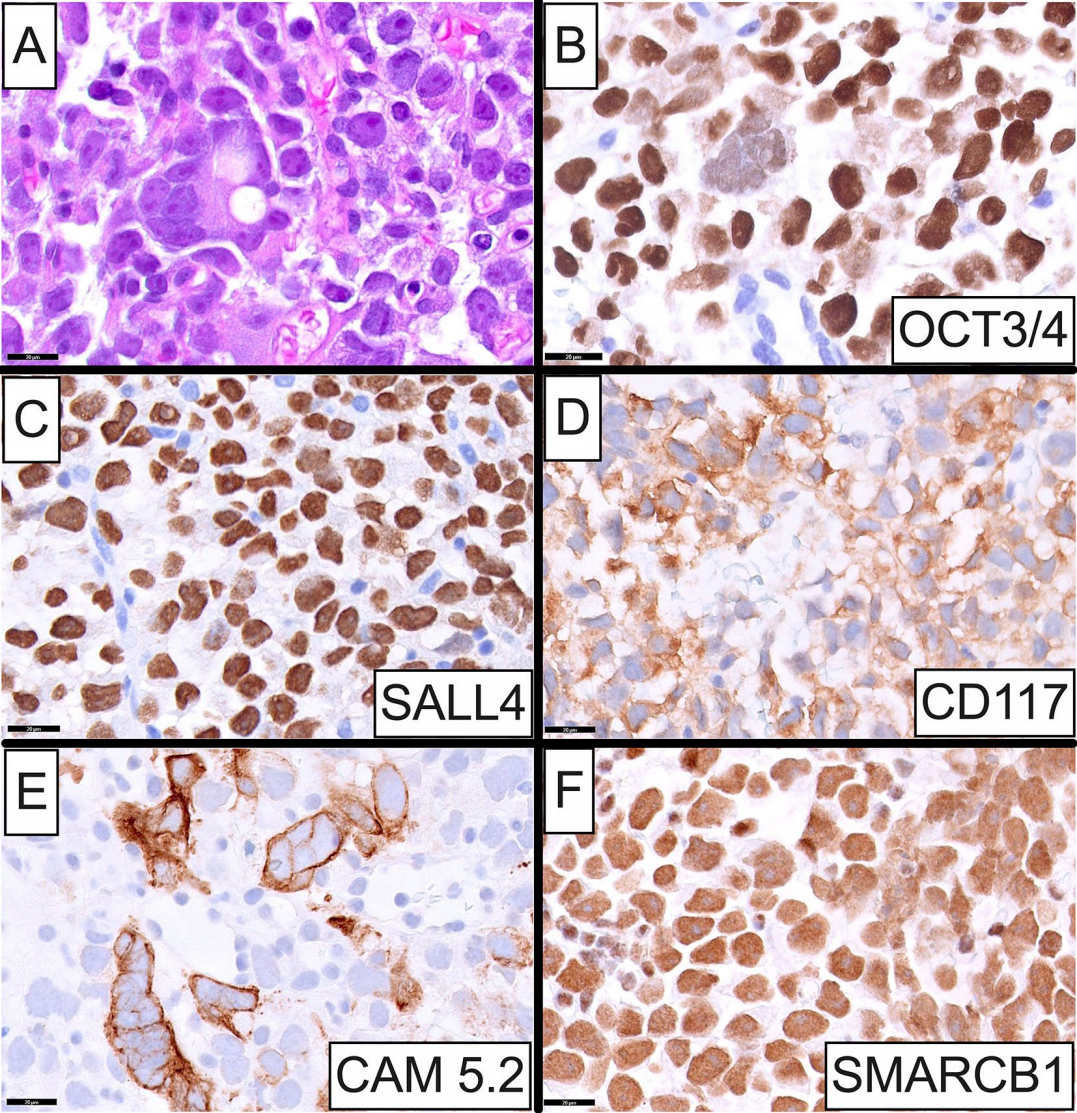
Histopathologic and immunohistochemical characteristics of the left supraclavicular lymph node. **A** High-power photomicrograph showing effacement of the normal nodal architecture and replacement by sheets of seminoma cells with interspersed syncytiotrophoblastic cells. The metastatic cells were diffusely positive for germ cell markers including OCT3/4 **B**, SALL4 **C** and CD117 **D**. CAM 5.2 **E** highlighted the syncytiotrophoblastic cells. In contrast to the somatic-type malignancy chondrosarcoma that was SMARCB1-deficient, nuclear SMARCB1 **F** expression was retained in the seminoma cells

Histopathologic examination of the enlarged left supraclavicular lymph node revealed effacement of the normal nodal architecture and replacement by sheets of seminoma cells with interspersed syncytiotrophoblastic cells (Fig. 4A). By IHC, these cells showed diffuse positivity for GCT markers including OCT3/4 (Fig. 4B), SALL4 (Fig. 4C), CD117 (c-KIT; Fig. 4D) and D2-40, corroborating the diagnosis of metastatic testicular seminoma, while CAM 5.2 (Fig. 4E), CK AE1/AE3, glypican 3 and β-HCG highlighted the syncytiotrophoblastic cells. Nuclear SMARCB1 expression was retained (Fig. 4F). The panel of negative IHC markers included SOX10, melan-A, S100, desmin, CD30, high molecular weight CK, CK MNF116 and EMA.

Given the discovery of a malignant TGCT and the patient’s disseminated metastatic lymphadenopathy, in conjunction with the unique immunophenotypic characteristics of the skull base cartilaginous neoplasm, i.e., loss of SMARCB1 expression with diffuse S100 and EMA positivity, and lack of *IDH1/2* mutations, the chondrosarcoma was interpreted as representing STM arising from the patient’s metastatic TGCT. Additional FISH studies revealed the presence of 1 to 4 copies of isochromosome 12p [i(12p)] in most metastatic nodal seminoma cells, while the chondrosarcoma cell population harbored duplication of 12p, thus corroborating the above diagnosis.

### Treatment and Follow-Up

The patient was treated with 4 cycles of combined BEP (bleomycin, etoposide, and cisplatin) chemotherapy. Three months after the initiation of treatment and while the patient was beginning his 4^th^ cycle of BEP, he presented with severe coughing, worsening dyspnea, and low oxygen saturation. Chest CT disclosed extensive pulmonary embolism of the right main pulmonary artery and ipsilateral middle and lower lobe vessels. Bronchoscopy followed by culture for microorganisms revealed coagulase-negative Staphylococci and Viridans Streptococci for which a cocktail of broad-spectrum antibiotics was administered. Due to suspected bleomycin-induced toxicity, the patient was also treated with increased dosage of prednisone. He developed pneumothorax and eventually succumbed to acute hypoxic and hypercarbic respiratory failure caused by acute respiratory distress syndrome and metastatic TGCT pulmonary emboli.

## Discussion

We report the clinicopathologic, immunophenotypic and molecular characteristics of a diagnostically challenging case of chondrosarcoma involving the nasal cavity and skull base of a man with previously undiagnosed malignant TGCT and extensive lymph node metastases. Several lines of evidence strongly indicate that the skull-based cartilaginous neoplasm represents a STM from metastatic TGCT. As we have shown, radiographically and histopathologically, the patient presented with widespread left metastatic nodal disease involving retroperitoneal, retrocrural, periaortic, mediastinal and, even, supraclavicular lymph nodes. Metastasis to the left supraclavicular (Virchow’s) node from infradiaphragmatic primary malignancies is, overall, considered uncommon [20, 21] and exceedingly rare in the case of TGCTs [22]. Therefore, intracranial and/or sinonasal TGCT metastasis is strongly favored given the presence of metastatic seminoma in supradiaphragmatic, including supraclavicular, lymph nodes.

Histopathologic diagnosis of STM arising within a primary or metastatic TGCT may be problematic. According to the most recent W.H.O. classification, criteria for the diagnosis of STM include an expansile overgrowth comprising a pure population of atypical mesenchymal or epithelial cells that occupy at least one low-power field (×4 objective, 5 mm in diameter) and show an infiltrative pattern [23]. Microscopically, STMs derived from TGCTs exhibit a markedly broad spectrum of histopathologic types lacking morphologic resemblance to TGCTs, including sarcomas (approximately 50% of all cases), carcinomas (20%), embryonic-type neuroectodermal tumor (10%), nephroblastoma and, even, myeloid neoplasms and undifferentiated tumors [14, 23, 24]. Notably, carcinomas and sarcomas occurring as TGCT STMs show divergent clinical characteristics, with the former developing later in the course of disease (median: 108 months post TGCT diagnosis) and the latter usually presenting earlier (median: 20 months) [13, 23]. Among the sarcoma subgroup, rhabdomyosarcoma accounts for greater than 50% of reported STM cases [11, 14, 18, 23].

Well-documented examples of TGCT with conventional chondrosarcoma as STM are sparse in the English literature and have been published either in the form of single case reports [9, 10] or as part of large-scale clinicopathologic cohorts [7, 8]. In the latter, STMs with chondrosarcomatous features account for just 3.7% [8] – 5% [7] of all TGCTs with STM. In all previously reported cases of STM chondrosarcoma (*N* = 5, age range = 19–53 years; age mean = 32.2 years), chondrosarcoma was discovered either within the primary testicular tumor [7, 9], or during retroperitoneal lymph node dissection [8], or both [10]. The present case is the first reported example of STM chondrosarcoma occurring in an extratesticular and extranodal anatomic location. Similar to most previous cases of TGCT with STM, chondrosarcomas arose within a pure teratoma [8, 10], mixed GCT with a teratomatous component [7, 9], or yolk sac tumor [7]. Even after extensive sampling, STMs may be lacking a recognizable GCT component [10, 14, 18, 25, 26], adding to the diagnostic complexity of such cases. In our patient, the primary testicular tumor and lymph node metastasis demonstrated features of seminoma with syncytiotrophoblastic cells, although no seminomatous component was identified in the vicinity of the chondrosarcoma. On rare occasions, STMs have been associated with “pure” seminomas [13, 17]. A plausible explanation for the above would be a sampling error during evaluation of the primary testicular tumor or foci of teratoma that escaped histologic detection due to their small size [17].

Another interesting finding regarding this chondrosarcoma, which further supports its etiopathogenesis as a STM from a metastatic TGCT, is its unique IHC properties. Although the overall histomorphologic characteristics of the tumor were unequivocally consistent with the diagnosis of chondrosarcoma, lesional cells showed strong and diffuse immunoreactivity for S100 and EMA, as well as focal staining with CAM 5.2 and CK AE1/AE3. Given that overt expression of epithelial differentiation markers is not anticipated in conventional chondrosarcoma [5, 27, 28], the observed S100 and EMA positivity raised suspicion for the diagnosis of chondroid chordoma. However, brachyury stain, a highly sensitive and specific marker for notochordal neoplasms [28, 29], was uniformly negative. Negativity was also observed for the GCT markers OCT3/4 and SALL4, which were strongly positive in the metastatic seminomatous cells. As reported previously [10, 14, 25], STMs commonly demonstrate a divergent immunoprofile compared to the TGCT of origin.

Genomic inactivation and loss of nuclear expression of SMARCB1, a core subunit of the SWItch Sucrose Non-Fermentable (SWI/SNF) chromatin remodeling complex, characterizes a plethora of mesenchymal neoplasms with, overall, diverse histopathologic appearance and biologic properties [30, 31]. SMARCB1-deficient soft tissue neoplasms traditionally comprise of a monomorphic population of undifferentiated epithelioid cells with “prototypical” rhabdoid cytomorphology, and anaplastic large-cell or small round-cell features [30, 32]. Multiple previous studies have shown that virtually all malignant rhabdoid tumors [33] and poorly-differentiated chordomas [34], 90% of epithelioid sarcomas [35, 36], as well as a subset of epithelioid malignant peripheral nerve sheath tumors [30, 31], myoepithelial carcinomas [35] and extraskeletal myxoid chondrosarcomas [37] demonstrate SMARCB1 inactivation and/or loss either by FISH or IHC. Furthermore, SMARCB1 germline mutations have been associated with familial schwannomatosis and meningiomatosis [33, 38], in which acquired NF2 mutations synergize with SMARCB1 inactivation [39]. Interestingly, the STM chondrosarcoma of the current patient exhibited uniform negativity for SMARCB1 by IHC in the sarcomatous cells but retained expression in non-neoplastic background cells. To the best of our knowledge, only one additional example of SMARCB1-deficient conventional chondrosarcoma has been previously reported and involved the mandible of a 13-year-old boy with a history of thoracic malignant rhabdoid tumor and an underlying germline SMARCB1 deletion [40]. The latter raised the question whether our patient also harbored a SMARCB1 germline aberration which would render him susceptible to the development of malignancies. However, as we showed, metastatic nodal seminoma cells were strongly positive for SMARCB1 by IHC, suggesting that loss of SMARCB1 in the STM chondrosarcoma represents an acquired genetic or epigenetic phenomenon rather than a germline event.

Early genetic alterations of *IDH1/2* have been identified in 46.1% [41] to 71.4% [42] and, more recently, in 85.7% [4] of skull base chondrosarcoma cases. *IDH1* mutations involving codon 132 of exon 4 predominate, with the R132C transition (CGT N TGT) detected in about 40% of the cases [43]. *IDH2* mutations are seen in 8.6% of chondrosarcomas, most of them being an R172S transversion (AGG N AGT) [43]. This chondrosarcoma was lacking *IDH1/2* mutations. Although a fraction of skull base chondrosarcomas may be negative for *IDH1/2* aberrations, it is also plausible that as a STM arising from a metastatic TGCT, this tumor is not driven by usual genetic mechanisms that underlie conventional chondrosarcomagenesis. Detection of i(12p) or overrepresentation of 12p is a hallmark feature of TGCTs and has been confirmed in most STMs [8, 44]. Therefore, cytogenetic studies for the identification of i(12p) by FISH [45, 46] or quantitative PCR may be diagnostically useful in cases of STM that are not associated with conventional GCT components [12, 46], like the current chondrosarcoma. Notably, FISH analysis confirmed abnormal duplication of 12p in the chondrosarcoma cells, as well as multiple copies of i(12p) in the metastatic seminoma cells, validating that the skull base chondrosarcoma represents STM arising from a metastatic seminoma.

Recognition of STMs arising in association with TGCTs is of utmost importance since their presence dramatically impacts prognosis and overall survival [14, 23]. Furthermore, proper identification of STM histopathologic subtypes may harbor therapeutic implications. Although cisplatin-based chemotherapy is highly effective for conventional TGCTs conferring excellent prognosis, such therapeutic regimens are often ineffective in STMs derived from TGCTs [13]. In agreement with that, our patient showed no signs of responsiveness to the administered cocktail of bleomycin, etoposide and cisplatin. Notwithstanding multimodality chemotherapeutic protocols, patients with STMs show cancer-specific survival rates ranging from 50 to 60% [14, 47].

In conclusion, herein we report a rare example of skull-based conventional chondrosarcoma in a 39-year-old man presenting as chemotherapy naive STM arising from a metastatic seminoma. Unlike “typical” cases of conventional head and neck chondrosarcoma, the STM chondrosarcoma showed loss of SMARCB1 expression, focal to diffuse immunostaining for epithelial markers, including EMA, CAM 5.2 and CK AE1/AE3, and 12p duplication with absence of *IDH1/2* mutations. Although exceedingly uncommon, metastasis to the head and neck region may occur in patients with testicular neoplasia. This case report also underscores the importance of high index of suspicion regarding the diagnosis of lesions that present with otherwise typical histomorphology but unexpected or uncommon IHC or molecular properties.

## Data Availability

All available clinical, histopathologic, immunohistochemical and molecular data pertaining to the current case are presented in the manuscript.
